# Prevalence of *Vibrio* spp. in Seafood from German Supermarkets and Fish Markets

**DOI:** 10.3390/foods13243987

**Published:** 2024-12-10

**Authors:** Christopher Zeidler, Vanessa Szott, Thomas Alter, Stephan Huehn-Lindenbein, Susanne Fleischmann

**Affiliations:** 1 School of Veterinary Medicine Center for Veterinary Public Health, Institute of Food Safety and Hygiene, Freie Universität Berlin, Königsweg 69, 14163 Berlin, Germany; christopher.zeidler@bht-berlin.de (C.Z.); vanessa.szott@fu-berlin.de (V.S.); thomas.alter@fu-berlin.de (T.A.); 2 Faculty of Life Sciences and Technology, University of Applied Sciences, Berliner Hochschule für Technik, Luxemburger Str. 10, 13353 Berlin, Germany; stephan.huehn-lindenbein@bht-berlin.de

**Keywords:** human pathogen *Vibrio* spp., prevalence, seafood, food safety, supermarkets, fish markets

## Abstract

This study investigates the prevalence of *Vibrio* spp. in seafood from supermarkets and fish markets in Berlin, Germany. A total of 306 seafood samples, including shrimp and mussels, were bought from supermarkets between March 2023 and January 2024. Samples were analysed using the ISO standard method and multiplex PCR to identify *V. parahaemolyticus*, *V. alginolyticus, V. cholerae* and *V. vulnificus*. The results indicated an overall *Vibrio* spp. prevalence of 56%. Among the positive samples, the most prevalent species found was *V. parahaemolyticus* (58%), followed by *V. alginolyticus* (42%), *V. cholerae* non-O1/non-O139 (25%), and *V. vulnificus* (4%). Samples obtained from supermarkets exhibited a lower prevalence (50%) than those received from fish markets (91%). Virulence genes such as *ctxA*, *tdh*, or *trh* were not detected in the respective *Vibrio* species. Nevertheless, the high prevalence underscores the need and urgency of continuous seafood surveillance.

## 1. Introduction

*Vibrio* (*V.*) species are halophilic and Gram-negative bacteria and occur ubiquitously in oceans, rivers, lakes and brackish water worldwide [1]. Climate change influences ocean temperatures, which in turn affects the growth conditions for various marine bacteria, including the *Vibrionaceae* family. Some species are recognized as human pathogens [2,3] and these changes already indicate that there is an increase in human cases of infection [4,5,6]. Given these facts and the steadily increasing seafood consumption, there is an urgent need for regular monitoring of the prevalence of pathogenic *Vibrio*. Moreover, the evolving situation has led to an increasing demand for the introduction of mandatory reporting for *Vibrio* cases in Germany, which has been implemented since 2020. Accordingly, the European Food Safety Authority (EFSA) recently underscored this necessity in their report, stressing the critical importance of consistent *Vibrio* surveillance in seafood. The importance of this study is enhanced by the EFSA report 2024, which recommends regular monitoring of *Vibrio* prevalences in seafood [7].

Overall, these bacteria are most frequently responsible for food-borne infections that are caused after the consumption of seafood containing *Vibrio* spp. Among the 12 *Vibrio* species identified as human pathogens, the 4 most common are *V. parahaemolyticus*, *V. alginolyticus, V. cholerae* and *V. vulnificus*. In humans, Vibriosis may manifest as watery diarrhea, abdominal pains, vomiting, and fever when contaminated water is ingested. The infection can also occur when raw or undercooked seafood (e.g., shrimp, mussels, or especially oysters) containing *Vibrio* is consumed [8,9,10]. However, clinical symptoms may vary depending on the route of infection, host susceptibility, and the specific pathogen involved [9]. Human pathogenic *Vibrio* spp. harbours a variety of virulence factors. *V*. *parahaemolyticus* contains the *trh* and *tdh* genes that encode for TDH-related hemolysin (TRH) and thermostable direct hemolysin (TDH), a main factor for its pathogenicity. These hemolysins are partially responsible for the pathogenicity of *V. parahaemolyticus* and induce lysis of erythrocytes by perforating the cell membrane, ultimately leading to cell death [9].

The most important representative *Vibrio* species is *V. cholerae,* which comprises more than 220 serogroups and is responsible for global cholera endemics and epidemics. The serogroups O1 and O139 are capable of expressing cholera toxin (CTX), which, in combination with toxin-coregulated pilus (TCP), constitute the virulence factors responsible for cholera. All other serotypes of *V. cholerae* are classified as non-O1/non-O139 *V. cholerae* but are also able to cause diarrheal diseases [1,2]. *V. alginolyticus* is primarily responsible for wound and ear infections [4,11] but it has also been described in food-associated intestinal infections [9]. However, so far, conclusive studies on foodborne infections caused by *V. alginolyticus* are scarce. It is interesting to note that *V. alginolyticus* is also responsible for infecting immunosuppressed marine species and is therefore of concern for industrial fish and seafood farming [9]. *V. vulnificus* often leads to severe wound infections. Furthermore, infections from eating seafood containing *Vibrio* spp. can occur, especially in immunocompromised individuals and those with chronic liver disease. Oysters containing *V. vulnificus* are responsible for food-related deaths in the USA [12].

The presence of *Vibrio* species in raw seafood is widespread, occurring in both wild and aquaculture environments across all types of seafood. Globally, *Vibrio* spp. is predominantly found in seafood from temperate climates. Due to their filter-feeding behavior, mussels actively accumulate microorganisms from the surrounding water in their digestive tract. Consequently, consuming raw oysters, for instance, is associated with an elevated risk of human *Vibrio* infection [1,13]. In shrimp, it is assumed that *Vibrio* can effectively colonize on the cilia and within the crevices, and has also been detected in the intestines of shrimp [14,15]. *Vibrio* can also be detected in fish, but with a lower prevalence of 13 to 33% [16,17,18].

In recent years, numerous studies have investigated the prevalence of *Vibrio* spp. in seafood and fish. Vu et al. [16], for example, reported a prevalence rate of 58% in shrimp and 53% in mussels from German retail in 2018. Furthermore, the authors determined significant differences in *Vibrio* spp. prevalence related to storage conditions: chilled and unpackaged seafood exhibited a higher prevalence rate (66%) than chilled and prepackaged seafood (36%) and frozen and packaged seafood (35%) [8]. Similarly, studies have indicated varying prevalence rates depending on the source and handling of seafood products. In Mexico, for instance, a prevalence of *Vibrio* spp. of 59% was found in shrimp at fish markets, with the majority of strains isolated from supermarkets (48%), followed by street vendors (37%) and retail stores (15%) [19].

Based on the described prevalence data, the rising number of infections, and climate change with its associated improved survival conditions, the aim of the present study was to provide an overview of the current prevalence of *Vibrio* spp. in seafood from German supermarkets and fish markets.

## 2. Materials and Methods

### 2.1. Sample Collection

The seafood examined in this study was collected from retail companies such as supermarkets and fish markets in Berlin, Germany, between March 2023 and January 2024. In total, 306 seafood samples were collected. Samples consisted of the following: black tiger shrimps (*Penaeus monodon*, *n* = 188), white-legged shrimp (*Litopenaeus vannamei*, *n* = 74), red shrimps (*Pleoticus muelleri*, *n* = 6), blue mussels (*Mytilus edulis*, *n* = 18), oysters (*Ostreidae* spp., *n* = 17) and scallop meat (*Pecten jacobaeus*, *n* = 3). Some of the seafood samples (*n* = 251) were obtained via self-service; hence the samples were packaged in an outer layer of paper with secondary packaging in plastic bags. The remaining seafood samples (*n* = 55) were obtained from seafood stores with a service counter. These seafood samples were stored on shaved ice alongside other seafood, and some had already undergone thawing. Subsequently, seafood samples were packed into plastic bags for transportation. Thereafter, all samples were kept in an isolated cool box with ice packs to maintain the cold chain and analysed within 2 h of purchase.

### 2.2. Isolation of Vibrio spp.

The presence of *V. parahaemolyticus*, *V. alginolyticus, V. cholerae* and *V. vulnificus* was determined according to the method described in EN ISO 21872-1:2017 [20] and ISO/TS 21872-2:2020 [21]. In brief, a 25 g sample was diluted with 225 mL alkaline peptone water (APW, Merck, Darmstadt, Germany) containing 2% NaCl and homogenized at 200 rpm for 60 s using a laboratory Smasher (bioMérieux, Durham, NC, USA). Samples were incubated for pre-enrichment for 6 h at 37 °C. Afterward, 1 mL of pre-enrichment dilution was transferred into 9 mL fresh APW and incubated for another 18 ± 1 h at 37 ± 1 °C. Subsequently, the enriched samples were streaked onto thiosulfate citrate bile sucrose agar (TCBS, Difco, BD, Le Pont de Claix, France) using 10 µL inoculation loops (Sarstedt, Nürnberg, Germany) and plates were incubated for 24 ± 1 h at 37 ± 1 °C. In addition to the TCBS agar described in the ISO method, *Vibrio* ChromoSelect Agar (VCS, Merck, Darmstadt, Germany) was used as an additional selective agar for further morphological confirmation of presumptive *Vibrio* spp. For subcultivation, typical presumptive *Vibrio* colonies were isolated from TCBS and VCS plates and assessed for the presence of cytochrome-c-oxidase using oxidase test strips (Bactident Oxidase, Merck, Darmstadt, Germany). Positive presumptive colonies were subsequently streaked onto lysogen broth agar (LB, Sigma-Aldrich, St. Louis, MO, USA). LB plates were then incubated for 24 ± 1 h at 37 ± 1 °C as well.

### 2.3. DNA Preparation

For DNA extraction, cytochrome c oxidase-positive colonies from pure cultures grown on LB agar were used. A single colony was transferred to a microcentrifuge tube containing 200 µL of sterile distilled water. DNA was thermally lysed for 10 min at 95 °C using a thermomixer (Eppendorf, ThermoMixer, Hamburg, Germany), followed by centrifugation at 17,530× *g* for 3 min. The supernatant was then carefully removed and stored at −20 ± 1 °C.

### 2.4. Multiplex PCR

Multiplex PCR (mPCR) based on a combined protocol of Bauer et al. [22] and Di Pinto et al. [23] was used to confirm and differentiate *V. parahaemolyticus*, *V. alginolyticus V. cholerae* and *V. vulnificus* (see Table 1). Process conditions were as follows: 30 amplification cycles, pre-denaturation at 94 ± 1 °C for 4 min, denaturation at 94 °C for 30 s, annealing at 64 °C for 30 s, extension at 72 °C for 30 s and a final extension at 72 °C for 7 min. The mPCR was performed with 2 µL (50 ± 10 ng/µL) of template and a total volume of 25 µL master mix. The gel electrophoresis was performed in a 3% agarose gel (Biozym Biotech, Vienna, Austria) at 120 V for 45 min. Visualization of gel bands, which present gen-specific PCR products of the target genes, was carried out using a UV light E-BOX CX5 TS chamber (Vilber, Collégien, France) and compared to those of reference strains (*V. parahaemolyticus* DSMZ 101031, *V. cholerae* DSMZ 101014, *V. alginolyticus* ATC 17749, *V. vulnificus* DSMZ 10143). To identify the presence of the *ctxA* gene in *V. cholerae* strains, a protocol by Shirai et al. [24] was used. The virulence genes *tdh* and *trh* in *V. parahaemolyticus* were detected by a PCR protocol described by Hossain et al. [25].

## 3. Results

In this study, 306 samples from various food matrices were analysed for the presence of *V. parahaemolyticus*, *V. alginolyticus*, *V. cholerae* and *V. vulnificus*. Of these samples, 56% (*n* = 170) were tested positive for at least one *Vibrio* species, while 44% (*n* = 136) were tested negative. The samples were distributed across different matrices such as shrimp (*n* = 268) and mussels (*n* = 38). No *Vibrio* species were detected in red shrimps (*Plesioteuthis muelleri*) with a sample size of 6. In contrast, blue mussels (*Mytilus edulis*), black tiger shrimp (*Penaeus monodon*), white-legged shrimp (*Litopenaeus vannamei*), scallop meat (*Pecten jacobaeus*) and oysters (*Ostreidae* spp.) demonstrated a *Vibrio* prevalence of 39% (*n* = 18), 54% (*n* = 188), 58% (*n* = 58), 67% (*n* = 3) and 100% (*n* = 17), respectively.

Presumptive colonies were determined based on their morphology on TCBS or VCS agar and identified as *V. parahaemolyticus*, *V. alginolyticus, V. cholerae* and *V. vulnificus* using multiplex PCR. When evaluating the prevalence across all samples examined, *V. parahaemolyticus* emerged as the most prevalent species with 58% (99/170), followed by *V. alginolyticus* with 25% (42/170), *V. cholerae* with 15% (25/170) and *V. vulnificus* with 2% (4/170). When comparing different matrices, *V. parahaemolyticus* was found in shrimps with a prevalence of 68% (98/144), while only a prevalence of 4% (1/26) was detected in mussels. Furthermore, *V. alginolyticus* showed the highest prevalence rate at 85% (22/26) in mussels. Regarding the place of purchase, the total prevalence of *V. parahaemolyticus*, *V. alginolyticus, V. cholerae* and *V. vulnificus* in supermarkets was found to be 50% (130/262), whereas seafood markets exhibited a prevalence of 91% (40/44). The most prevalent species in supermarkets was *V. parahaemolyticus* with 71% (92/130), whereas in seafood markets, *V. alginolyticus* showed the highest prevalence with 70% (28/40) (see Table 2).

Among all *Vibrio* positive samples, the majority harboured only one *Vibrio* species with 74% (126/170), while a smaller proportion 26% (44/170) contained more than one *Vibrio* species. In addition, there were three samples that contained three *Vibrio* species and one sample in which all four *Vibrio* species examined were detected (Supplementary Table S1). Regarding virulence genes, the *ctxA* gene could not be detected in any of the *V. cholerae* isolates (*n* = 90). The *tdh* and *trh* genes were not detected in the examined *V. parahaemolyticus* isolates (*n* = 365), respectively.

## 4. Discussion

Rising seafood consumption and changing climate conditions favouring *Vibrio* growth are expected to increase *Vibrio*-related foodborne infections [7]. The aim of the present study was to provide a comprehensive overview of the current prevalence of *Vibrio* spp. in seafood from German supermarkets and fish markets. For this reason, a total of 306 seafood samples were collected and analysed according to EN ISO 21872-1:2017 [20] and ISO/TS 21872-2:2020 [21] standard methods and multiplex PCR to identify *V. parahaemolyticus*, *V. alginolyticus, V. cholerae* and *V. vulnificus*.

### 4.1. Total Prevalence

For the subsequent discussion of the results, we accounted for differences in sample size, methodology, and food matrices in other prevalence studies to enable a direct comparison of prevalence rates. The results of the conducted study reveal an overall prevalence for *V. parahaemolyticus*, *V. alginolyticus, V. cholerae* and *V. vulnificus* of 54% (170/306). In accordance, Vu et al. determined a prevalence rate of 55% (88/160) in seafood samples [8]. Comparable results have been observed in other studies focusing primarily on shrimps and mussels. For instance, a study from Morocco found a prevalence of 56% (167/299) in shrimp [14] while Hirshfeld et al. reported a prevalence rate of 60% (241/400) in a study conducted in the USA [27]. Nevertheless, prevalence rates may vary widely across different regions around the world. In Italy, for example, authors reported a prevalence rate of 7.6% (33/435), while research groups from Sri Lanka and Ecuador observed much higher rates of 95% (162/170) and 96% (219/229), respectively [28,29,30]. It can be assumed that these varying prevalence rates are likely due to different import routes for seafood. In Germany, only 21% of the seafood consumed is produced locally, with the majority being imported from countries where pathogenic *Vibrio* spp. is endemic. In general, these countries have stable warm water temperatures, which create optimal conditions for the proliferation of *Vibrio* spp. [12]. As global water temperatures continue to rise, both the abundance and the species composition of *Vibrio* are affected [1,3].

In this study, multiple *Vibrio* spp. were detected in 26% (44/170) of the seafood samples examined, while single species were identified in the majority of the seafood samples (74% (126/170)). Other studies that identified multiple species reported prevalence rates of 15% in Germany [8], 21% in Sri Lanka [29] and 46% in Ecuador [30]. Prevalence discrepancies may be attributed to factors such as cross-contamination [16,27]. In addition, the diversity of *Vibrio* spp. is determined by certain fishing areas, salinity and water temperature [1,7]. As the water temperature rises, the abundance of *Vibrio* spp. increases [3]. In fact, Vibriosis cases have increased globally, even in colder regions. In the U.S. for example, infections rose by 109% in 2018 compared to 2015–2017, due to climate change, rising sea levels and changes in salinity, which are increasing *Vibrio* incidence and thus the disease incidence worldwide [31].

### 4.2. Prevalence of V. parahaemolyticus

In the current study, *V. parahaemolyticus* emerged as the most dominant species, accounting for 58% of all samples examined. This species was particularly prevalent in shrimps with a prevalence of 68%. In other studies, it is suspected that the wide distribution in aquatic environments worldwide is the cause of the high prevalence of *V. parahaemolyticus* [32]. Regarding growth conditions, *V. parahaemolyticus* (NaCl range: 0.5–10%, optimum 0.5%) shows a significantly broader range of salt tolerance compared to *V. cholerae* (NaCl range: 0.1–4.0%, optimum 3.0%), which likely explains its global distribution [7]. This fact likely contributes to its status as a major cause of seafood-borne gastroenteritis associated with the consumption of raw or undercooked products [15]. Indeed, this particular species is a common pathogen found in seafood around the world [33].

Additionally, several studies in China have described the prevalence of *V*. *parahaemolyticus* in shrimps ranging from 33% to 84% [18,34,35,36,37,38]. Notably, in Sri Lanka, the prevalence was particularly high with 91% (155/170). Controversially, authors in Morocco reported a significantly lower prevalence of *V. parahaemolyticus* in locally harvested shrimp at 8.4% (25/299), while the prevalence of *V. alginolyticus* was much higher at 54% (161/299) [14]. A study by Sterk et al. determined the predicted concentration of *Vibrio* spp. with increasing water temperatures due to climate change. Worst-case scenarios were investigated in which the water surfaces of different Dutch waters rise by 2.3 and 3.7 °C. Under future climate scenarios, concentrations of *Vibrio* spp., including *V. parahaemolyticus*, are expected to rise. However, *V. parahaemolyticus* shows a smaller increase compared to *V. alginolyticus* due to lower temperature sensitivity [39]. The influence of temperature on the growth conditions of *Vibrio* spp. was also described in the North and Baltic Sea [3]. As mentioned above, salinity of the water can significantly influence the prevalence of *V. parahaemolyticus* as well [1].

Regarding the presence of *tdh* or *trh* genes, in this study, none of the isolates were found encoding these genes. To date, no tdh-positive *V. parahaemolyticus* isolates have been reported in seafood originating from German coastal waters [12,40]. Furthermore, *V. parahaemolyticus* strains harbouring the *tdh* gene are rarely detected in imported seafood in Germany. This is shown by a study in 2022 that revealed a prevalence rate of 60% for *V. parahaemolyticus* (543/902) with no detection of the *tdh* gene. However, strains carrying the *trh* gene are observed with moderate frequency, ranging from 3.0% to 5.0%, in North European coastal areas [12]. In agreement, Kang et al. reported that 54% (38/71) of isolates from oysters were tested positive for the presence of the *trh* gene, whereas none contained the *tdh* gene [41]. However, it should be noted that the virulence factors of *V. parahaemolyticus* are complex and not fully understood. It is assumed that the hemolysin TDH and/or TRH are not necessarily the only virulence factors of pathogenic *V. parahaemolyticus* isolates [32,42].

### 4.3. Prevalence of V. alginolyticus

The prevalence of *V. alginolyticus* in this study was found to be 25%. Comparative data from previous research indicate varying rates: 10% (10/100) in Egypt, 19% (32/170) in Sri Lanka, 36% in Germany and 50% in Ecuador [8,16,29,30]. Some researchers assume that *V. alginolyticus* acts as a commensal in shrimps and plays an important role in shrimp farms as a utilizer of the unused, accumulated feed. In general, *Vibrio* spp. is considered capable of degrading chitin [14]. This hypothesis is further supported by a study conducted by Hirshfeld et al. who observed a higher prevalence of *Vibrio* spp. in farmed shrimp (78% (211/269)) than in wild-caught shrimp (46% (60/131)) [27]. In contrast, in our study, a comparatively low prevalence of *V. alginolyticus* was determined in shrimp (14%), while its prevalence in mussels was notably higher (85%). Since mussels feed by filtering water, they also accumulate pathogens from their aquatic environment in their gut, which could explain the higher prevalence of *Vibrio* spp. [43]. This observation is consistent with studies from the German Wadden Sea, where a similarly high prevalence of *Vibrio* spp. in mussels of 71% (61/82) was determined. In this study, the most commonly detected species was *V. alginolyticus*, with a prevalence of 51%, followed by *V. parahaemolyticus* (39.5%), *V. cholerae* (4.7%) and *V. vulnificus* (3.5%) [44]. Huehn et al. also reported similar prevalence and the same order of occurrence of *Vibrio* species in a review in which the results of a study of mussels from the Wadden Sea in Lower Saxony were evaluated. Furthermore, multiple *Vibrio* species were frequently found in a single sample, with a clear seasonal pattern. While *V. alginolyticus* was present throughout the year, the other three species were detected exclusively during the summer and autumn [1]. Around 80% of the mussels sold in German retail originate from the European Union. The largest mussel farms operate in Germany, Denmark, the Netherlands, the UK and Ireland and are located in the cool-temperate climate zone [12]. It is described that the number of mussels contaminated with *V. parahaemolyticus* and *V. vulnificus* decreases with decreasing water temperatures, which could explain the dominance of *V. alginolyticus* in terms of prevalence [12,44].

### 4.4. Prevalence of V. cholerae and V. vulnificus

In this study, *V. cholerae* was detected in 15%, whereas *V. vulnificus* was found in 2.2% of the samples. In contrast, much lower prevalence rates of *V. cholerae* were observed in Sri Lanka, with 4.1% (7/170), and in Senegal, with 1.6% (2/123) [29,45]. It is noteworthy that none of the *V. cholerae* isolates examined carried the *ctxA* gene.

One reason for the low prevalence of *V. vulnificus* could be its high temperature sensitivity [44]. Despite the relatively low prevalence of 2.2%, this result is concerning given that *V. vulnificus* infections exhibit an extremely high lethality rate (40 to 60%), especially in immunocompromised individuals or those suffering from liver or stomach problems [46]. In particular, oysters harvested in the USA have been linked to fatal *V. vulnificus* infections in these vulnerable groups [12]. In this study, however, *V. vulnificus* was not detected in any of the oysters examined (0/17). Notably, *V. vulnificus* was detected in 2.6% (4/144) of the shrimp samples. It is important to highlight that infections caused by *V. vulnificus* are relatively rare in Germany, suggesting that not all strains of this pathogen are equally virulent. The genetic diversity of *V. vulnificus* is substantial, which contributes to a varied virulence potential among different strains [12].

### 4.5. Differences Between Supermarkets and Seafood Markets

The type of packaging appears to influence the prevalence of *Vibrio* spp. In this study, packed samples from supermarkets showed a prevalence of 50% (130/262), while loose-sold samples from seafood markets exhibited a higher prevalence of 91% (40/44). In China, comparable trends have been observed: the prevalence of *V. parahaemolyticus* in crustacea received from wet markets was reported to be 86% (53/62), compared to 73% (19/26) in supermarkets [38]. The notably higher prevalence in loose-sold samples compared to packaged samples may reflect differences in food processing and transportation. Cross-contamination could be a significant factor contributing to the elevated prevalence of *Vibrio* spp. in unpacked food. Indeed, some authors suggest that direct placement of seafood on ice alongside other products may enhance the risk of cross-contamination [8,9].

### 4.6. Methods Used and Their Limitations

For the detection of *Vibrio* species, various methods have been established. Conventional cultivation relies either on direct plating on selective or non-selective agar media or on pre-enrichment in alkaline peptone water followed by plating on selective agar. The methods used in this study were based on internationally used ISO standards, as these methods are well established, validated and verified. However, cultivation-based methods also have limitations. For instance, the characterization of pathogenicity based on virulence genes is optional. Additionally, randomly isolating a limited number of colonies may potentially result in underestimating the prevalence of pathogenic strains in the examined samples [7]. During enrichment, a dominant strain or species often outcompetes others, rendering it challenging to detect multiple species. Furthermore, it is also difficult to determine the initial number of bacteria because enrichment is performed. A further limitation of the methods used for the study is the potential of *Vibrio* to enter the viable but n-culturable (VBNC) state. In this state, they are metabolically active but can no longer be cultivated on standard culture media [3,7,12]. Thereby, the bacteria are able to survive and retain their virulence.

In this study, *V. parahaemolyticus*, *V. alginolyticus*, *V. cholerae* and *V. vulnificus* were identified and confirmed using PCR methods. However, as Bonny et al. noted, the choice of method should depend on the specific objectives of the investigation. Conventional PCR is simple, sensitive, cost-effective, and requires only small sample volumes, while mPCR allows simultaneous detection of multiple genes. Nevertheless, it should be noted that these methods are unable to differentiate between living and dead bacteria and carry a risk of non-specific amplification in mPCR [47].

## 5. Conclusions

This study provides a comprehensive overview of *Vibrio* spp. prevalence in seafood from German supermarkets and fish markets. In total, *Vibrio* spp. was present in 54% (170/306) of the samples examined. The most frequently detected species was *V. parahaemolyticus* (58%, 99/170), followed by *V. alginolyticus* (25%, 42/170), *V. cholerae* non-O1/O139 (15%, 25/170) and *V. vulnificus* (2.2%, 4/170). Furthermore, multiple *Vibrio* spp. were detected in 26% (44/170) of the seafood samples examined. Notably, loose-sold seafood products from seafood markets exhibited a higher *Vibrio* spp. prevalence (91%) than packaged products from the supermarket (50%), indicating potential cross-contamination issues. Importantly, no virulence genes such as *ctxA*, *tdh*, or *trh* were detected in the respective *Vibrio* species. However, it should be noted that isolates can also cause infections without these toxins. In this study, conventional cultivation methods were performed according to ISO standards including enrichment in alkaline peptone water and cultivation on TCBS and VCS agar. PCR methods were used to confirm *V. parahaemolyticus* (*tdh*, *trh*), *V. alginolyticus*, *V. cholerae* (*ctxA*), *V. vulnificus* and its toxins, respectively. While reliable, these methods have limitations, such as underestimating pathogenic strains due to dominance during enrichment and inability to detect viable but non-culturable (VBNC) bacteria. Despite these limitations, the methods are robust but require careful selection to meet specific study goals.

In summary, this study emphasizes the urgent need for stringent surveillance and enhanced hygiene practices to mitigate or reduce the contamination of seafood with *Vibrio* spp. Future research should focus on identifying potential sources of cross-contamination and national tracking trends in *Vibrio* spp. prevalence as environmental conditions evolve. The results obtained will contribute to the monitoring of the presence of these pathogenic bacteria in seafood sold directly to end consumers, especially as increasing global temperatures favour the growth conditions of *Vibrio* spp.

## Figures and Tables

**Table 1:** Primers used for multiplex PCR.

Target Gene	5′-3′ Primer Sequence	Reference	Amplicon Size (bp)
*V. parahaemolyticus UtoxF*	GASTTTGTTTGGCGYGARCAAGGTT	[22]	297
*V. parahaemolyticus vptoxR*	GGTTCAACGATTGCGTCAGAAG		
*V. cholerae UtoxF*	GASTTTGTTTGGCGYGARCAAGGTT	s.a.	640
*V. cholerae vctoxR*	GGTTAGCAACGATGCGTAAG		
*V. vulnificus UtoxF*	GASTTTGTTTGGCGYGARCAAGGTT	s.a.	435
*V. vulnificus vvtoxR*	AACGGAACTTAGACTCCGAC		
*V. alginolyticus VA-F*	CGAGTACAGTCACTTGAA AGCC	[23]	737
*V. alginolyticus VA-R*	CACAACAGAACTCGCGTTACC		
*V. cholerae ompW1-F*	CACCAAGAAGGTGACTTTATTGTG	[26]	588
*V. cholerae ompW2-R*	CTCAGACGGGATTTGTTAGGCACG		
*V. cholerae ctxA1-F*	CTCAGACGGGATTTGTTAGGCACG	[24]	301
*V. cholerae ctxA2-R*	TCTATCTCTGTAGCCCCTATTACG		
*V. parahaemolyticus GRO-1-F*	AGGTCAGGCTAAGCGCGTAAGC	[25]	510
*V. parahaemolyticus GRO-2-R*	GTCACCGTATTCACCCGTCGCT		
*V. parahaemolyticus TDH-1-F*	TATCCATGTTGGCTGCATTCAAAAC	s.a.	171
*V. parahaemolyticus TDH-2-R*	TCTTCACCAACAAAGTTAGCTACA		
*V. parahaemolyticus TRH-1-F*	TTCAACGGTCTTCACAAAATCAGA	s.a.	382
*V. parahaemolyticus TRH-2-R*	AAACATATGTCCATTTCCGCTCTC		

s.a. see above.

**Table 2:** Prevalence of *V. parahaemolyticus*, *V. alginolyticus, V. cholerae* and *V. vulnificus* in total, in seafood and in places of purchase.

*Vibrio* Species ^a^	Prevalence									
	Prevalence of Samples in Total (%)/No. ^b^ (*n* = 170)		Prevalence in Shrimps (%)/No. ^b^ (*n* = 144)		Prevalence in Mussels (%)/No. ^b^ (*n* = 26)		Prevalence in Supermarkets (%)/No. ^b^ (*n* = 130)		Prevalence in Fish Markets (%)/No. ^b^ (*n* = 40)	
*V. parahaemolyticus*	58	(99)	68	(98)	3.8	(1)	71	(92)	18	(7)
*V. alginolyticus*	25	(42)	14	(20)	85	(22)	11	(14)	70	(28)
*V. cholerae*	15	(25)	15	(22)	12	(3)	16	(21)	11	(5)
*V. vulnificus*	2.2	(4)	2.6	(4)	0	(0)	2.5	(3)	1.3	(1)

^a^ Overall prevalence from samples with multiple detected species; ^b^ values in parentheses are the number of positive samples.

## Data Availability

The original contributions presented in the study are included in the article and Supplementary Material, further inquiries can be directed to the corresponding author.

## Supplementary Materials

The following supporting information can be downloaded at https://www.mdpi.com/article/10.3390/foods13243987/s1: Table S1: Prevalence of single and multiple *Vibrio* species in seafood in total, in shrimp, in mussels and in different places of purchase.
