# Supplementary material for: Prevalence of Vibrio spp. in Seafood from German Supermarkets and Fish Markets
Source: Foods. 2024 Dec 10;13(24):3987. doi: 10.3390/foods13243987 (PMC11675752; doi:10.3390/foods13243987)
Supplement: Supplementary file 1 [file foods-13-03987-s001.zip › foods-3300462-supplementary.pdf]

Table S1. Prevalence of single and multiple *Vibrio* species in seafood in total, in shrimp, in mussels and in different places of purchase

Combination *Vibrio* species	Prevalence of samples in total (%)/ No. [a], (n= 170)		in shrimp (%)/ No. [a], (n= 144)		in mussels (%)/ No. [a], (n= 26)		in super-market (%)/ No. [a], (n= 130)		in seafood markets (%)/ No. [a], (n= 40)	
V. parahaemolyticus	48	(81)	56	(81)	0	(0)	58	(76)	13	(5)
V. alginolyticus	19	(32)	8	(12)	77	(20)	5	(7)	63	(25)
V. cholerae	6	(11)	7	(10)	4	(1)	7	(9)	5	(2)
V. vulnificus	1	(2)	1	(2)	0	(0)	2	(2)	0	(0)
V. alginolyticus + *V. vulnificus*	1	(2)	1	(2)	0	(0)	2	(2)	0	(0)
V. cholerae + *V. parahaemolyticus*	12	(21)	14	(20)	4	(1)	15	(20)	3	(1)
V. vulnificus + *V. parahaemolyticus*	1	(1)	1	(1)	0	(0)	0	(0)	3	(1)
V. alginolyticus + *V. parahaemolyticus*	6	(11)	7	(10)	4	(1)	7	(9)	5	(2)
V. alginolyticus + *V. cholerae*	3	(5)	1	(2)	12	(3)	1	(1)	10	(4)
V. vulnificus + *V. parahaemolyticus* + *V. cholerae*	2	(3)	2	(3)	0	(0)	2	(3)	0	(0)
V. vulnificus + *V. parahaemolyticus* + *V. cholerae* + *V. alginolyticus*	1	(1)	1	(1)	0	(0)	1	(1)	0	(0)

[a] Values in parentheses are the number of positive samples
